# Photographic assessment of urostomy characteristics after ileal conduit diversion: a feasibility study

**DOI:** 10.1007/s00345-026-06544-5

**Published:** 2026-06-26

**Authors:** Joëlle M. Posthumus, M. W. van de Kamp, L. Fransen, B. W. G. van Rhijn, K. Hendricksen, H. G. van der Poel, E. Wit, J. B. Ringia, P. A. de Bie Leuveling Tjeenk, L. S. Mertens

**Affiliations:** Department of Urology, Antoni Van Leeuwenhoek, Amsterdam, The Netherlands

**Keywords:** Urostomy, Photographic characteristics, Radical cystectomy, Ileal conduit

## Abstract

**Purpose:**

A well-functioning urostomy is essential for quality of life after radical cystectomy with ileal conduit diversion. However, objective external characteristics of a well-functioning urostomy remain poorly defined. This feasibility study investigated whether postoperative photographs can be standardized and used to assess relevant urostomy characteristics.

**Methods:**

This single-centre study included 49 patients who underwent radical cystectomy with ileal conduit between June 2022 and December 2022. Immediate postoperative photographs were analysed. Urostomy characteristics, i.e. location, morphology, colour and height were evaluated by two experts using predefined criteria. Photographic standardization was reviewed by a medical photographer.

**Results:**

Of 49 patients, a total of 89 photographs were available; 49 overview (55%), 31 close-up (35%), and 42 side-view (47%). Urostomy location, colour, and morphology were assessable in 82–88% of cases. Mesentery location was assessable in only 48% and lacked reproducibility. Height and size could not be measured due to absence of a scale. Inter-observer agreement between the two experts and the wound, ostomy and continence nurse (WOC nurse) was 100% on whether a characteristic could be assessed in a photograph, with no discrepancies in scoring. The medical photographer identified substantial variability in lighting, angle, and composition and provided recommendations for standardization.

**Conclusion:**

Photographic documentation of urostomies is feasible and allows for reliable assessment of several characteristics. Standardization is essential for reproducibility. Future studies should correlate standardized photographic characteristics with functional outcomes to define determinants of a well-functioning urostomy.

## Background and objective

Radical cystectomy with urinary diversion has been the standard treatment for patients with muscle-invasive and very-high risk non-muscle invasive bladder cancer (BC) [1]. An ileal conduit is the most common incontinent urinary diversion. Despite its widespread use, more than half of patients experience urostomy-related complications, with considerable impact on quality of life [2–6].

**Fig. 1:**
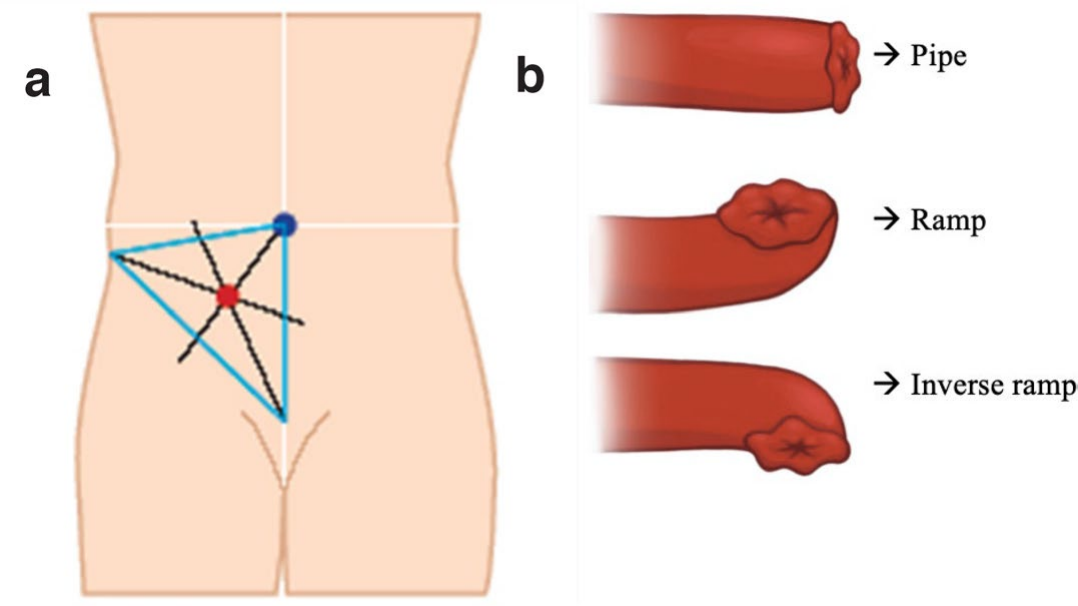
**a** The urostomy triangle. Preferred location for urostomy placement based on the anatomical landmarks umbilicus, pubis, and anterior superior iliac spine (ASIS). **b** Urostomy morphology subtypes: pipe, ramp, and inverse-ramp

Strategies to optimize urostomy outcomes have primarily focused on preoperative stoma site marking. Several reports, supported by guidelines and expert consensus, recommend positioning the urostomy within the so-called ‘urostomy triangle’ [7–11] (Fig. 1a) while avoiding skin folds [12, 13]. Placement through the rectus abdominis muscle may further reduce the risk of parastomal herniation and improve appliance adherence [14, 15].

Beyond stoma location, several external urostomy characteristics may theoretically influence stoma function and complications. Although evidence specific to ileal conduit urostomies is limited, studies in colorectal stomas suggest that insufficient stoma protrusion (< 2 cm) may be associated with leakage and peristomal skin complications [7, 19], whereas excessive protrusion may cause discomfort, particularly in women [20, 21].

Similarly, mucosal colour and moisture are commonly used as indicators of stoma vitality [16–18].

Morphological subtypes (pipe, ramp, inverse ramp; Fig. 1b) have been described, though their clinical impact remains unclear. Furthermore, mesenteric location may theoretically influence leakage risk, yet this has not been systematically studied.

Despite these hypotheses, objective characteristics of a well-functioning urostomy remain poorly defined. Furthermore, contemporary literature describing urostomy construction and assessment is scarce, and developments in this field appear to have been limited over the past decade [22]. Standardized documentation is a prerequisite for comparative studies. Photographic assessment may offer a simple and reproducible method for objective documentation, but its feasibility has not previously been evaluated. Therefore, the aim of this study was to assess the feasibility of standardized postoperative photographic documentation of urostomies and to determine which urostomy characteristics can be reliably evaluated using photographs.

## Methods

### Study design and population

This single-center feasibility study included all patients undergoing radical cystectomy with ileal conduit urinary diversion at the Netherlands Cancer Institute between June 2022 and December 2022. Surgical approaches included robot-assisted cystectomy, open cystectomy, total pelvic exenteration, and conversion from neobladder to ileal conduit. Urostomies were constructed according to institutional practice, and preoperative site marking was performed by specialized Wound, Ostomy and Continence nurses (WOC nurses). Patients without available photographs were excluded. All patients provided consent for the use of medical data for research. The Institutional Review Board (IRB) approved the study (IRBd23-096).

### Photography and data collection

Immediate postoperative images of urostomies were obtained in the operating room at the discretion of the surgeon, without specifications or instructions. Three types of photographs were available: overview images showing the abdominal wall and anatomical landmarks, close-up images focusing on the urostomy itself, and side-view images allowing assessment of urostomy protrusion and morphology. Among the 49 included patients, a total of 89 photographs were available, including 49 overview photographs, 31 close-up photographs, and 42 side-view photographs.

All photographs were stored in the electronic patient record, linked to the date of surgery, and retrieved by the investigator (J. M. P.) for analysis. Photographs were independently reviewed by the investigator and an experienced WOC nurse using predefined assessment criteria. In addition, a professional medical photographer reviewed all photographs with regard to image quality and standardization. Patient demographics and clinical data were retrieved from medical records.

### Urostomy characteristics

The primary outcome was feasibility of standardized photographic assessment of urostomy characteristics. Predefined characteristics were selected based on literature, guidelines, and expert consensus. Data were entered into an anonymized CASTOR EDC database.

Predefined urostomy characteristics for photographic assessment included:

- Location within the urostomy triangle, defined by the umbilicus–pubis–ASIS landmarks (Fig. 1a**)** [12, 13].
- Presence within a skin fold (yes/no) [12, 13].
- Mesenteric location, assessed as visible or not visible and, when visible, categorized as cranial or caudal.
- Height and size, defined as distance above the skin in centimeters and urostomy diameter, measurable only when a scale is present [7, 19–21].
- Colour assessed as visible or non-visible and, when visible, categorized as red, dark red/purple, or black [16–18].
- Morphology, categorized as pipe, ramp, or inverse ramp morphology (Fig. 1b).
- Peristomal skin condition, assessed using the SACS classification [7, 21].

Assessment was performed according to a structured framework. For each predefined characteristic, photographs were first evaluated for visibility and assessability. Characteristics that could be reliably identified were subsequently scored according to predefined criteria. Overview photographs were used to assess anatomical location and the presence of skin folds, close-up photographs were used to assess colour and mesenteric location, and side-view photographs were used to assess morphology. Colour was categorized as red and moist, dark red–purple, or black based on the overall visual appearance of the urostomy. As no validated classification system for urostomy colour is currently available, this classification was based on clinical assessment of mucosal vitality.

The selected characteristics were chosen because of their potential association with stoma function, leakage, appliance adherence, and peristomal skin complications. Because evidence specific to ileal conduit urostomies is limited, several characteristics, including stoma height, were derived from the colorectal stoma literature.

### Standardization of urostomy photography

A professional medical photographer reviewed all available photographs and provided recommendations for a standardized photography protocol. The review focused on image composition, camera angle, lighting conditions, camera settings, and the use of measurement scales. Based on this evaluation, recommendations for future standardized image acquisition were developed.

## Results

### Patient characteristics

Of 61 eligible patients, 49 had postoperative photographs available and were included (Table 1). The reason for the missing photographs is unknown. Mean age was 64.6 years, and 37% were female. Most underwent robot-assisted cystectomy (76%).

**Table 1:** Baseline demographic, clinical, and surgical characteristics of the study population (n = 49)

Characteristics, *n* = 49	*n* (%)
Female	18 (37)
Age mean (SD), years	64.6 (10)
BMI mean (SD), kg/m^2^	25.1 (4)
Comorbidities	
Smoker	8 (16)
Former smoker	23 (47)
Diabetes mellitus	6 (12)
COPD	3 (6)
Heart failure	1 (2)
Peripheral arterial disease	1 (2)
ASA score 1	4 (8)
ASA score 2	34 (69)
ASA score 3	11 (22)
Type of surgery	
Robot-assisted cystectomy	37 (76)
Open cystectomy	8 (16)
Total exenteration	3 (6)
Conversion from neobladder to ileal conduit	1 (2)

*SD* standard deviation; BMI, body mass index; *COPD* Chronic Obstructive Pulmonary Disease; *ASA* American Society of Anesthesiologists Physical Status, *n* number of patients

### Objective assessment of urostomy characteristics

**Fig. 2:**
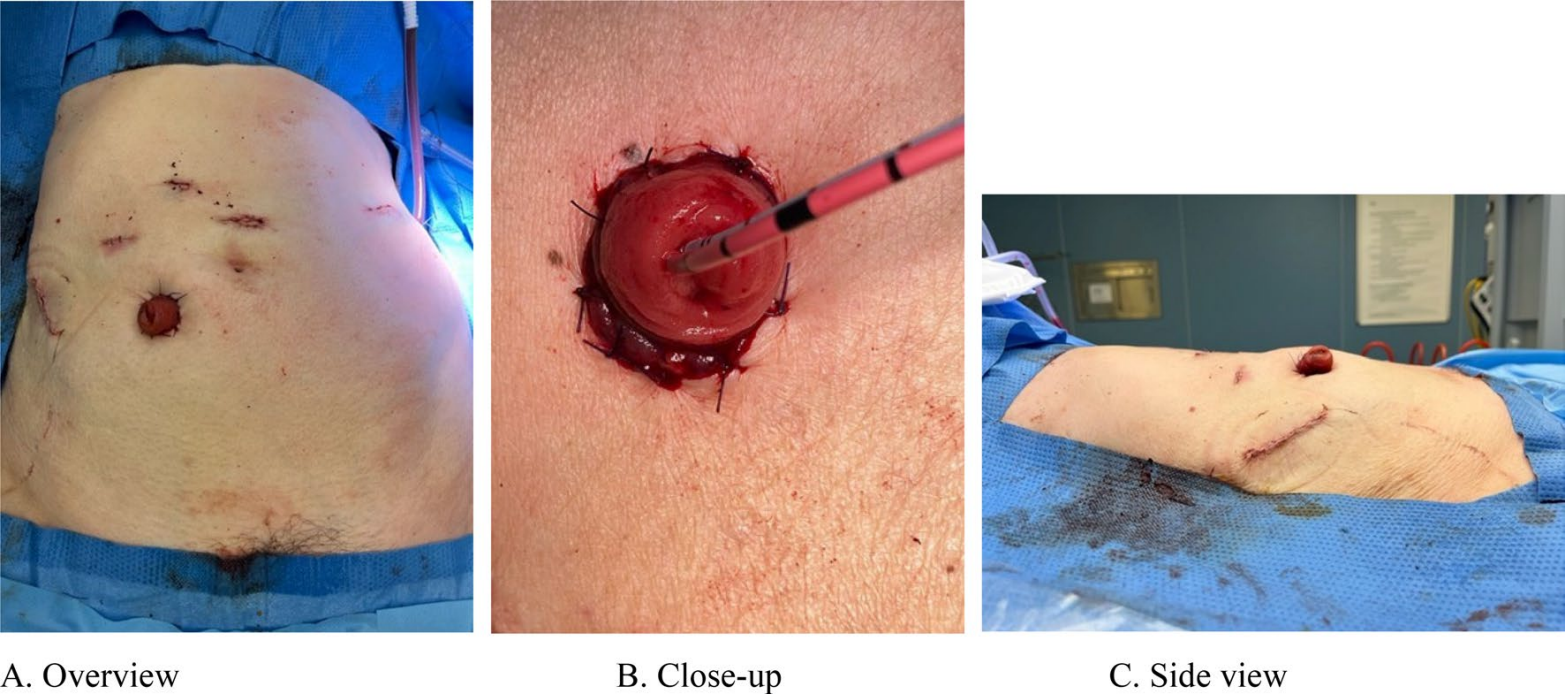
Standard photographic views used for urostomy assessment. **A** Overview photograph showing anatomical landmarks and urostomy location. **B** Close-up photograph for assessment of colour and mesenteric orientation. **C** Side-view photograph for assessment of protrusion and morphology

A total of 89 photographs were available; comprising 49 overview photographs (55%), 31 close-up photographs (35%), and 42 side-view photographs (47%) (Fig. 2 A–C).

Assessability of urostomy characteristics varied according to the characteristic evaluated and the type of photograph available (Table 2). Urostomy location within the predefined anatomical triangle was assessable in 56% of overview photographs. Assessment of skin folds was possible in 22 cases; none of the evaluated urostomies were located within a skin fold. Mesenteric location could be assessed in 16 of 33 photographs (48%). Among assessable cases, the mesentery was located in the cranial aspect of the urostomy in 75%. However, reproducibility of mesenteric location assessment was limited. Colour was assessable in 33 photographs, of which 88% were categorized as red and moist and 12% as dark red–purple. No urostomies were classified as black. Morphology was assessable in 82% of cases and categorized as pipe (48%), ramp (37%), or inverse-ramp (15%). Objective measurement of urostomy height and diameter was not possible because no measurement scale was present in any of the photographs.

**Table 2:**
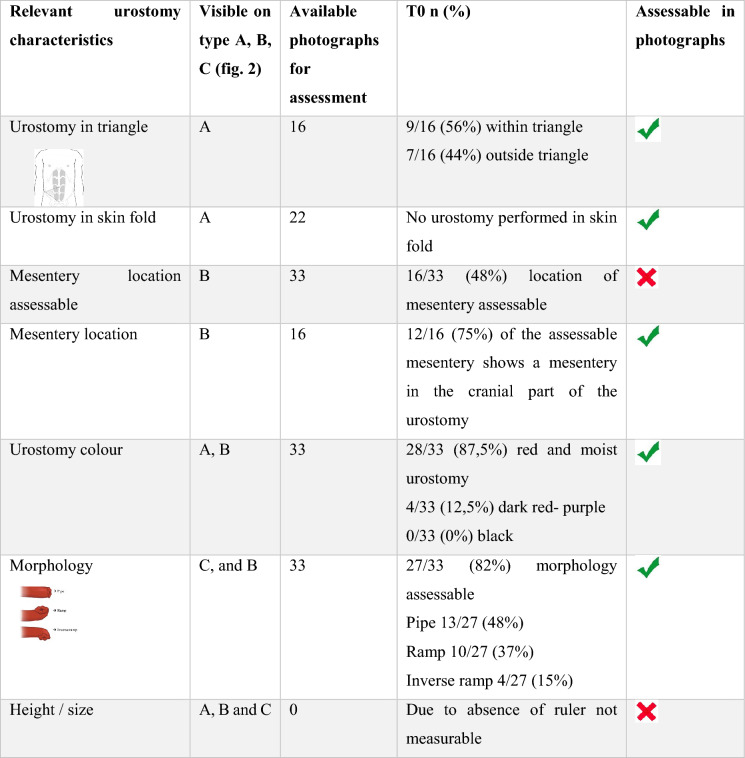
Assessability and findings of predefined urostomy characteristics on postoperative photographs

Agreement regarding assessability of the predefined characteristics was 100% between the investigator and the WOC nurse. No discrepancies in classification were observed for characteristics that could be assessed.

### Standardizing urostomy photography

Review by a professional medical photographer demonstrated substantial variability in image acquisition, including differences in zoom, camera angle, composition, and lighting. In addition, none of the photographs contained a measurement scale, precluding objective assessment of urostomy dimensions.

Based on this evaluation, a standardized photography protocol was proposed. Key recommendations included acquisition of three predefined photographic views (overview, close-up, and side-view), standardized lighting conditions, uniform camera settings, inclusion of a ruler or measurement scale, and documentation of mesenteric orientation during surgery.

## Discussion

This feasibility study demonstrates that postoperative photographs can be used to assess several urostomy characteristics, including location, colour, and morphology. Inter-observer agreement was excellent, supporting the reproducibility of photographic assessment when characteristics were visible. In contrast, mesentery location was inconsistently assessable, and objective measurement of height and diameter was not possible because no measurement scale was present in any of the photographs.

The most consistently assessable characteristic was urostomy location. Preoperative stoma site marking has been associated with improved patient satisfaction and reduced complication rates [7–11]. In the present study, preoperative stoma site marking was performed by a WOC nurse in all patients. According to the operative records, none of the surgeons changed the stoma site intraoperatively from the location preoperatively marked by the WOC nurse. Despite variability in photographic quality, it was always possible to determine whether the urostomy was positioned within the predefined anatomical triangle [13]. This suggests that photographs may provide a practical method for documenting urostomy location and evaluating adherence to existing recommendations regarding stoma placement.

Assessment of skin folds was feasible, although limited by the supine positioning of patients at the time of photography. Upright images may reveal folds not visible in the operating room and could better reflect real-life conditions of stoma care. Colour was assessable in the majority of cases and a red, moist mucosa is considered a proxy for adequate vascular supply [16–18]. Although colour assessment remains subjective and no validated classification system is currently available, photographic documentation may provide a useful method for longitudinal monitoring of urostomy vitality.

Morphology was also reliably assessed. Although the clinical relevance of the morphology subtypes remains uncertain, inverse ramp morphology has been hypothesized to predispose to leakage by directing urine toward the peristomal skin. The ability to classify morphology in more than 80% of cases supports the feasibility of future studies investigating potential associations between morphology and functional outcomes. In contrast, mesenteric location proved difficult to evaluate and lacked reproducibility, even when potentially visible. These findings suggest that intraoperative documentation may be more reliable than photographic assessment for this characteristic.

An important observation was the variability in photographic technique. The review by the medical photographer demonstrated differences in zoom, angle, and lighting, as well as the absence of a scale in all photographs. These findings underscore that technical aspects of image acquisition in an important determinant of assessability and reproducibility.

Based on this evaluation, a structured photography protocol is proposed to facilitate standardized documentation of urostomies across centres and over time. The protocol aims to minimize variation in image acquisition and ensure that all relevant urostomy characteristics can be evaluated consistently across patients, centres, and time points. At each assessment time point, three predefined photographic views should be obtained for every patient: an overview photograph demonstrating anatomical landmarks and urostomy location, a close-up photograph for assessment of colour and mesenteric orientation, and a side-view photograph for assessment of protrusion and morphology.

### Proposed standardized photography protocol

#### Lighting

- Turn off spotlights in the operating room.
- Prevent shadows on photographs.
- Use additional illumination when required to ensure adequate image quality.
- Do not combine artificial and natural light sources.
- Prevent backlighting.

#### Choice of camera

- Use a single high-quality camera system.
- Use the same camera system during follow-up assessments whenever possible.
- Use standardized camera settings throughout the study.
- Standardize white balance settings.
- Choose and consistently apply a single image format (RAW or JPEG).

#### Structured photographs

- Obtain all three predefined photographic views (overview, close-up, and side-view) for every patient at each assessment time point.
- Use the same patient posture for all photographs.
- Include a ruler or measurement scale in all photographs.
- Archive photographs according to predefined image categories (overview, close-up, and side-view).
- Record mesenteric orientation intraoperatively, as this characteristic cannot be reliably assessed from photographs alone.

To our knowledge, this is the first study to systematically assess the feasibility of standardized photographic assessment of urostomy characteristics following ileal conduit diversion. Strengths include the novel methodology, involvement of both a WOC nurse and a professional medical photographer, and the use of predefined assessment criteria. Limitations include the small sample size, single-center design, and restriction to immediate postoperative photographs, which may not reflect long-term changes in urostomy appearance resulting from resolution of oedema, postoperative healing, or weight changes. Colour assessment was based on visual interpretation and no objective colour calibration method was available. Finally, the cohort included different surgical procedures. Although most patients underwent robot-assisted radical cystectomy, a minority underwent open cystectomy, total pelvic exenteration, or conversion from a neobladder to an ileal conduit. Although all urostomies were constructed according to the same institutional principles and assessed using identical criteria, differences in operative approach may influence urostomy appearance and cannot be excluded.

Future studies should prospectively implement a standardized photography protocol at predefined postoperative time points and correlate photographic characteristics with clinically relevant outcomes, including leakage, peristomal skin complications, appliance wear time, stoma-related interventions, and patient-reported quality of life. Such a longitudinal design may help identify which urostomy characteristics are associated with optimal long-term function and determine whether early postoperative appearance predicts later outcomes.

In conclusion, photographic documentation of urostomies is feasible and allows reliable assessment of several external characteristics. Standardization of image acquisition is essential to improve reproducibility and enable objective assessment. Prospective studies incorporating standardized photography and longitudinal clinical outcomes are now warranted to define the characteristics of a well-functioning urostomy. Such a longitudinal approach may help define the determinants of optimal urostomy performance and assess whether early postoperative appearance predicts long-term outcomes.

## Data Availability

All data supporting the findings of this study are available within the paper. The specific photographs are not publicly available due to privacy reasons. The data are, however, available upon request and with the permission of Antoni van Leeuwenhoek Hospital.
